# Exploring the use of tablet computer-based electronic data capture system to assess patient reported measures among patients with chronic kidney disease: a pilot study

**DOI:** 10.1186/s12882-017-0771-7

**Published:** 2017-12-06

**Authors:** Dorothy Wong, Shen Cao, Heather Ford, Candice Richardson, Dmitri Belenko, Evan Tang, Luca Ugenti, Eleanor Warsmann, Amanda Sissons, Yalinie Kulandaivelu, Nathaniel Edwards, Marta Novak, Madeline Li, Istvan Mucsi

**Affiliations:** 1 Division of Nephrology, Multi-Organ Transplant Program, University Health Network and University of Toronto, Toronto, Canada; 2 Centre for Mental Health, University Health Network and Department of Psychiatry, University of Toronto, Toronto, Canada; 3 Department of Supportive Care, Princess Margaret Hospital, Toronto, ON Canada; 4 Multi-Organ Transplant Program, Toronto General Hospital, University Health Network, 585 University Avenue, 11-PMB-188, Toronto, ON M5G 2N2 Canada

**Keywords:** Patient reported outcome measures (PROMs), Tablet computers, CKD, Diabetes, Electronic data capture, Self-administered questionnaires, Pilot study

## Abstract

**Background:**

Collecting patient reported outcome measures (PROMs) via computer-based electronic data capture system may improve feasibility and facilitate implementation in clinical care. We report our initial experience about the acceptability of touch-screen tablet computer-based, self-administered questionnaires among patients with chronic kidney disease (CKD), including stage 5 CKD treated with renal replacement therapies (RRT) (either dialysis or transplant).

**Methods:**

We enrolled a convenience sample of patients with stage 4 and 5 CKD (including patients on dialysis or after kidney transplant) in a single-centre, cross-sectional pilot study. Participants completed validated questionnaires programmed on an electronic data capture system (DADOS, Techna Inc., Toronto) on tablet computers. The primary objective was to evaluate the acceptability and feasibility of using tablet-based electronic data capture in patients with CKD. Descriptive statistics, Fischer’s exact test and multivariable logistic regression models were used for data analysis.

**Results:**

One hundred and twenty one patients (55% male, mean age (± SD) of 58 (±14) years, 49% Caucasian) participated in the study. Ninety-two percent of the respondents indicated that the computer tablet was acceptable and 79% of the participants required no or minimal help for completing the questionnaires. Acceptance of tablets was lower among patients 70 years or older (75% vs. 95%; *p* = 0.011) and with little previous computer experience (81% vs. 96%; *p* = 0.05). Furthermore, a greater level of assistance was more frequently required by patients who were older (45% vs. 15%; *p* = 0.009), had lower level of education (33% vs. 14%; *p* = 0.027), low health literacy (79% vs. 12%; p = 0.027), and little previous experience with computers (52% vs. 10%; p = 0.027).

**Conclusions:**

Tablet computer-based electronic data capture to administer PROMs was acceptable and feasible for most respondents and could therefore be used to systematically assess PROMs among patients with CKD. Special consideration should focus on elderly patients with little previous computer experience, since they may require more assistance with completion.

**Electronic supplementary material:**

The online version of this article (10.1186/s12882-017-0771-7) contains supplementary material, which is available to authorized users.

## Background

Chronic kidney disease (CKD) is a major public health concern that leads to poor health outcomes and substantial disease burden for patients and families. In the past decade, an extended body of studies have highlighted the alarming rate of psychosocial distress within the CKD population, with the prevalence of depression ranging between 14 and 30% in patients with stage 5 CKD [1] and anxiety detected in up to 51% of patients on maintenance dialysis [2, 3]. Depression not only has a negative impact on health-related quality of life [4, 5], but is also associated with poor treatment adherence, hospitalization [6], high morbidity [4] and overall mortality [4, 5, 7–9]. Despite these concerning facts, systematic assessment of symptoms (including mental health comorbidities) in the clinical management of CKD is not implemented as a standard of care, and depression and anxiety is often unrecognized and undertreated [10, 11].

There is a growing interest in the use of patient-reported outcome measures (PROMs) for psychosocial distress screening [12], symptom monitoring and management [12], as well as risk stratification among patients with chronic medical conditions [8, 13–15]. PROMs include physical symptom lists [16, 17], illness intrusiveness [18–21], treatment decision making [22–25], social support [26–32], psychosocial distress [33–35] as well as health-related quality of life [36]. Assessment of the patient’s own perception on functional and psychosocial well-being, quality of life and social support needs, however, is rarely incorporated in routine nephrology practice [37, 38]. This could be done using validated standard questionnaires to regularly collect PROMs to capture various dimensions of the patient’s subjective experience.

Electronic data capture systems offer a sustainable and economical way for the routine assessment of PROMs. Using electronic data capture instead of paper-based questionnaires to assess PROMs may improve the feasibility of assessing PROMs in routine clinical practice. It eliminates the need for subsequent data entry, storage of the questionnaires and reduces the risk of privacy breach. It has the potential for immediate scoring and presentation of results [39–41], offers the potential to link PROMs with clinical data in electronic health records [42, 43], enhance communication in multidisciplinary care [44, 45], and facilitate the assessment of PROMs independently from patient provider encounters. Electronic capture of PROMs has been utilized in oncology and palliative care [39, 40, 44, 46]. Schick-Makaroff et al. has shown that there was overall satisfaction with electronic capture of patient reported outcomes among patients receiving home hemodialysis [47, 48]. Computer literacy, however, varies within different patient populations, and less educated, elderly patients may face barriers when offered to use tablet-based questionnaires. Establishing the tablet’s feasibility among patients with CKD is an important first step in incorporating routine use of “ePROMs” in clinical practice.

The purpose of the current pilot study was to assess the acceptability of electronic touch-screen tablet computers in collecting PROMs among patients with advanced CKD, including stage 5 CKD treated with renal replacement therapies (either dialysis or transplant).

## Methods

### Study population

This is a single-centre, cross-sectional pilot study of patients with CKD at the University Health Network, Toronto, Canada. We recruited a convenience sample of 121 participants from the nephrology and kidney transplant outpatient clinics, in-centre and home hemodialysis units and the kidney transplant in-patient unit between September 2015 and April 2016. Individuals who were 18 years or older with stage 4 or 5 chronic kidney disease (eGFR < 29 mL/min/1.76m^2^ and eGFR < 15 mL/min/1.76m^2,^ respectively) were eligible, including those with stage 5 CKD treated with renal replacement therapies (either dialysis or transplant). eGFR was determined by the CKD-EPI Equation. Patients who have been on dialysis treatment for more than 90 days have been recruited from the dialysis center of our hospital; in their case eGFR was not assessed as an inclusion criterion. Similarly, kidney transplant recipients by definition have stage 5 CKD(T), therefore eGFR was not used to select those patients either. Finally, pre-dialysis patients were recruited from the “Renal Management Clinic” of our hospital. This clinic provides complex, multidisciplinary management for patients with stage 4 and 5 CKD who are at significant risk of their CKD progressing. Patients are referred to this clinic for modality education and preparation for RRT from general nephrology clinics, where they had been followed for some time. Consequently, the chronic nature of CKD has been well established for all study participants.

Individuals with diagnosis of dementia, severe acute medical conditions, or unable to understand and read English were excluded. The study was approved by the University Health Network Research Ethics Board.

Participants were asked to complete sets of questionnaires on tablet computer devices. The research staff was present at all times to demonstrate the use of the tablet device or help with completing the questionnaires if needed.

### Questionnaires

In this study, standard, validated questionnaires were programmed on an electronic data capture system (DADOS, Techna Inc., Toronto). In order to reduce respondent burden, the total questionnaire pool was divided into 4 sets (A - D) each containing a different combination of questionnaires (Table 1). To report the acceptability and the amount of assistance needed for completing the questionnaires, participants answered six questions that provided information about their experience with completing the questionnaires on the tablet computers (Patient Response Questionnaire - PRQ) (Additional file 1). The following questionnaires were used:

**Table 1:** Questionnaire items and sets used in pilot study

Questionnaire Items	Set A	Set B	Set C	Set D
Transplant Decision Making Survey (TDMS) [22]	X			
Distress Assessment and Response Tool (DART) [48]		X	X	
Experience in Close Relationship Scale (ECR) [27]	X			
Relationship Questionnaire (RQ) [30, 31]	X	X	X	
Short Literacy Survey (SLS) [50]	X	X	X	
MOS Social Support [32]				X
Illness Intrusiveness Rating Scale (IIRS) [18–21]		X	X	
Kidney Disease Quality of Life Questionnaire [36]				X
Patient Response Questionnaire	X	X	X	X
Socio-demographic Questionnaire	X	X	X	X
Estimated average completion time	42 min	32 min	32 min	33 min

#### Patient response questionnaire (PRQ)

The PRQ was designed to assess participants’ reaction to computer tablets. Participants were asked to complete 6 questions, which explored: 1) tablet acceptability, 2) help required in completing the tablet questionnaire, 3) questionnaire acceptability, 4) whether task of tablet completion was tiring, 5) participant’s previous computer experience, and 6) level of comfort in using computer tablet technology.

**Transplant Decision Making Survey (TDMS)** provides a validated, theoretically consistent measure of 1) Stages of Change, a measure of readiness to pursue deceased and living donor kidney transplant; 2) Decisional Balance, a weighted assessment of the pros and cons of deceased and living donor kidney transplant (DDKT and LDKT); and 3) Self-Efficacy, a measure of patient confidence in pursuing DDKT or LDKT in difficult circumstances [22]. These measures are based on the Transtheoretical Model of Behaviour Change [23], which has been successfully applied to assess transplant decision making [24, 25].

**Distress Assessment and Response Tool (DART)** [49] .consists of validated instruments for assessing psychosocial distress: 1) Patient Health Questionnaire-9 (PHQ-9) for depression [33, 34]; 2) Generalized Anxiety Disorder-7 item (GAD-7) for anxiety [35], 3) Edmonton Symptom Assessment System-revised (ESAS-r) to assess symptom burden [16, 17], 4) the Social Difficulties Inventory (SDI) for practical problems [26]; and 5) the Canadian Problem Checklist (CPC) for problems frequently encountered by patients with chronic disease [50]. DART has been implemented as part of routine clinical care in the cancer center of our hospital [49]

**Experiences in Close Relationship Scale - Short Form (ECR-Short Form)** is used to assess adult attachment style. The ECR yields validated scales for attachment anxiety and attachment avoidance [27–29].

**Relationship Questionnaire (RQ)** consists of four brief paragraphs describing the four adult attachment patterns, including secure, dismissing, preoccupied and fearful. The four attachment patterns are rated on a 7-point Likert scale [30, 31].

**Short Literacy Survey (SLS)** is composed of three self-reported screening questions that are effective in predicting low and moderate subjective health literacy in both general medicine and surgical clinics [51]**.**

**Medical Outcomes Study (MOS) Social Support Survey** assesses various dimensions of social support, including emotional/informational, tangible, affectionate support, and positive social interaction. The 19 items on the survey are rated on a Likert scale [32].

**Illness Intrusiveness Rating Scale (IIRS)** measures the perceived intrusiveness of chronic disease and its treatment into one’s valued life domains. The theoretical framework has been tested, and the questionnaire has been validated in patients with different medical conditions, including CKD [18–21]^.^

**Kidney Disease Quality of Life Questionnaire-Short Form (**KDQOL-SF™) has been the most widely used quality of life measure for patients with renal diseases. The KDQOL-SF™ includes the Medical Outcomes Study Short Form-36 generic core (SF-36) and several multi-item scales targeted at quality of life concerns of special relevance for patients with CKD [36].

Socio-demographic characteristics, including age, gender, family composition, education level, employment status and ethno-cultural background were also collected from the participant. Clinical characteristics, including etiology of kidney disease, comorbidities, duration of CKD and dialysis modality were collected from paper charts and electronic medical records.

### Exposure variables

The exposure variables considered in the analysis includes age, gender, ethnicity, income, education, previous experience with computer technology, and self-reported health literacy. These variables were collected through socio-demographics, short literacy survey, and patient response questionnaires, and were selected for analysis based on both clinical experience and current literature.

### Outcome variables

The three outcome variables were collected from the patient response questionnaire: 1) “Did you find the task of completing the questionnaires on the tablet computer acceptable” (yes/no); 2) “Did you need someone’s help to complete the questionnaire” (none – little/some - a lot); and 3) “Did you find the task of completing the questionnaires on the tablet computer too difficult or tiring” (yes/no).

### Statistical analysis

All statistical analyses were performed using STATA 14.0 (StataCorp, College Station, TX). All results were described using appropriate descriptive statistics. Categorical variables were summarized using frequencies and percentages, while continuous variables were described using mean and standard deviation (SD).

Fischer’s exact test was employed to examine univariable associations between categorical exposure and outcome variables. Age was dichotomized using 70 years as cut-off. Self-reported income was used to describe socio-economic status, and categorized into low-middle (≤$70,000) and middle-high (>$70,000), based on the 2015 Ontario Income Tax Bracket. Education level was divided into low (none to high school diploma) and high (college degree or more). Multivariable associations between the exposure and outcome were explored using logistic regression models. Given the small number of patients who found the electronic data capture not acceptable or tiring, we used the “Penalized Maximum Likelihood Estimation” method proposed by Firth to reduce the consequent small-sample bias [52]. A two-sided *P* value of <0.05 was considered statistically significant in all analyses.

## Results

**Fig. 1:**
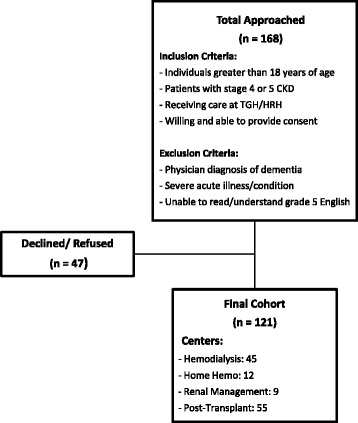
Flow Diagram of Patient Recruitment from September 2015 to April 2016

We approached a convenience sample of 168 potentially eligible patients, of whom 121 consented to participate, with an overall response rate of 72% (Fig. 1). The demographic and clinical characteristics of the study participants were summarized in Table 2. Mean (±SD) age was 58 (±14) years, 55% were male, and 49% were Caucasian. The most common cause of end stage renal disease was hypertension (37%), followed by glomerulonephritis (31%), and diabetes mellitus (26%). Seven percent of the patients were in the pre-dialysis stage, 47% were undergoing maintenance hemodialysis, while 45% were transplant recipients. Seventy four percent of participants reported average or above average experience with computer technology, and only a small fraction of participants answered “uncomfortable” (12%) when it comes to comfort with tablet technology. The average time needed to complete one of the four questionnaire sets (Table 1) varied between 25 and 45 min.

**Table 2:** Demographic and clinical characteristics of study sample

Variable	Total *N* = 121	Pre-Dialysis *N* = 9	Dialysis *N* = 57	Transplant *N* = 55
Mean Age; mean (SD)	58 (14)	60 (11)	60 (13)	54 (14)
Male; n (%)	67 (55)	4 (44)	29 (51)	34 (62)
Ethnicity; n (%)				
Caucasian	59 (49)	7 (78)	24 (42)	28 (51)
African Canadian	24 (20)	1 (11)	14 (25)	9 (16)
Asian	19 (16)	1 (11)	10 (18)	8 (15)
Other/Unknown	19 (16)	0	9 (16)	10 (18)
Marital status; n (%)				
Single	26 (21)	1 (11)	11 (19)	14 (25)
Married	69 (57)	6 (67)	28 (49)	35 (64)
Widowed, divorced or separated	25 (21)	1 (11)	18 (32)	6 (11)
Education; n (%)				
Low (≤ 12 yrs)	39 (33)	2 (22)	18 (33)	19 (35)
High (> 12 yrs)	78 (67)	7 (78)	36 (67)	35 (64)
Income; n (%)				
Low-Middle (≤$70,000)	57 (47)	5 (56)	27 (47)	25 (45)
Middle-High (>$70,000)	33 (27)	2 (22)	10 (18)	21 (38)
Unknown	31 (26)	2 (22)	20 (35)	9 (16)
Cause of ESKD; n (%)				
Glomerulonephritis	37 (31)	4 (44)	12 (21)	21 (38)
Diabetes Mellitus	31 (26)	1 (11)	16 (28)	14 (25)
Polycystic Kidney Disease	11 (9)	1 (11)	4 (7)	6 (11)
Hypertension	45 (37)	6 (67)	25 (44)	14 (25)
Computer Experience; n (%)				
Low	31 (26)	0	21 (37)	10 (18)
Average	44 (36)	3 (33)	14 (25)	27 (49)
High	46 (38)	6 (67)	22 (39)	18 (33)
Comfort with Computer Technology; n(%)				
Uncomfortable	11 (12)	0	7 (16)	4 (9)
Good	32 (34)	1 (14)	18 (42)	13 (29)
Excellent	52 (55)	6 (86)	18 (42)	28 (62)

Overall, the vast majority, 92% of participants found the task of completing the questionnaires on the tablet acceptable (Table 3). Lower levels of acceptance was observed among participants of older age (older vs. younger than 70 years; 75% vs. 95%; *p* = 0.011) and among the ones with little previous experience with computers (81% v. 96%; *p* = 0.050). In multivariable analysis, only previous computer experience (OR 6.19, 95% CI 1.06, 36.21) was significantly associated with acceptance of the electronic data capture systems (Table 6).

**Table 3:** Participant response on “Did you find the task of completing the questionnaires on the tablet computer acceptable”

Variables; n (%)	Yes 110 (92)	No 10 (8)	*P* value
Age			
≤ 70 years	95 (95)	5 (5)	0.011*
> 70 years	15 (75)	5 (25)	
Gender			
Male	61 (91)	6 (9)	1.000
Female	49 (92)	4 (8)	
Ethnicity			
White	55 (93)	4 (7)	0.743
Non-White	55 (90)	6 (10)	
Income			
Low (≤$70,000)	51 (89)	6 (11)	0.082
High (>$70,000)	33 (100)	0	
Education			
Low (≤ 12 yrs)	36 (92)	3 (8)	1.000
High (> 12 yrs)	72 (92)	6 (8)	
Health Literacy			
Low	12 (92)	1 (8)	1.000
Moderate	55 (93)	4 (7)	
High	29 (94)	2 (6)	
Computer Experience			
Low	25 (81)	6 (19)	0.050*
Average	41 (95)	2 (5)	
High	44 (96)	2 (4)	

(*) denotes statistically significance at p= <0.05

**Fig. 2:**
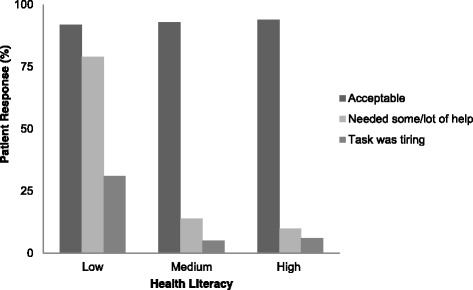
Association between health literacy and 1) finding the task of completing the questionnaires on tablet computer acceptable; 2) requiring help to complete the questionnaire; and 3) finding the task of completing questionnaires too difficult or tiring. Low health literacy was associated with needing more help with completion (*p* < 0.001) and finding the task too tiring or difficult (*p* = 0.019)

**Fig. 3:**
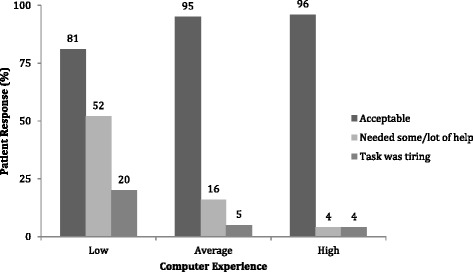
Association between computer experience and 1) finding the task of completing the questionnaires on tablet computer acceptable; 2) requiring help to complete the questionnaire; and 3) finding the task of completing questionnaires too difficult or tiring. Low computer experience is associated with lower acceptance (*p* = 0.05), needing more help with completion (p < 0.001), and finding the task too tiring or difficult (*p* = 0.037)

Overall, the majority of patients required none to very little help (79%) with the completion of questionnaires on electronic data capture system (Table 4). Greater level of assistance was more frequently required by patients who were older (45% vs. 15%; *p* = 0.009), had lower income (30% vs. 3%; *p* < 0.001), as well as lower levels of education (33% vs. 14%; *p* = 0.027). Moreover, patients with lower health literacy (Fig. 2) and less computer experience (Figure 3) also required more help with tablet use. In multivariable analysis, low health literacy (OR 8.65, 95% CI 1.77, 42.40) and low level of previous experience with computers (OR 5.17, 95% CI 1.34, 19.97), but not age, income, or education, were significantly associated with higher need for assistance (Table 6).

**Table 4:** Participant response on “Did you need someone’s help to complete the questionnaire”

Variables n (%)	None-Little 96 (79)	Some - A Lot 25 (21)	*P* value
Age			
≤ 70 years	85 (84)	16 (16)	0.009*
> 70 years	11 (55)	9 (45)	
Gender			
Male	54 (81)	13 (19)	0.437
Female	42 (78)	12 (22)	
Ethnicity			
White	50 (85)	9 (15)	0.181
Non-White	46 (74)	16 (26)	
Income			
Low (<$70,000)	40 (70)	17 (30)	<0.001*
High (>$70,000)	32 (97)	1 (3)	
Education			
Low (≤ 12 yrs)	26 (67)	13 (33)	0.027*
High (> 12 yrs)	67 (86)	11 (14)	
Health Literacy			
Low	3 (21)	11 (79)	<0.001*
Moderate	51 (86)	8 (14)	
High	28 (90)	3 (10)	
Computer Experience			
Low	15 (48)	16 (52)	<0.001*
Average	37 (84)	7 (16)	
High	44 (96)	2 (4)	

(*) denotes statistically significance at p= <0.05

A great majority of patients (92%) indicated that they did not find the task of completing tablet questionnaires tiring (Table 5). Participants with low income (12% vs. 0%; *p* = 0.044), low health literacy (31% vs. 6%; *p* = 0.019) and low levels of computer experience (20% vs. 4%; *p* = 0.037) were more likely to find the task difficult or tiring. However, these univariable associations did not reach significance after adjustment in multivariable models (Table 6).

**Table 5:** Participant response on “Did you find the task of completing the questionnaires on the tablet computer too difficult or tiring”

Variables n (%)	Yes 10 (8)	No 110 (9)	*P* value
Age			
≤ 70 years	8 (8)	93 (92)	0.658
> 70 years	2 (11)	17 (89)	
Gender			
Male	7 (10)	60 (90)	0.509
Female	3 (6)	50 (94)	
Ethnicity			
Caucasian	2 (3)	56 (97)	0.097
Non-Caucasian	8 (13)	54 (87)	
Income			
Low (<$70,000)	7 (12)	50 (88)	0.044*
High (>$70,000)	0 (0)	33 (10)	
Education			
Low (≤ 12 yrs)	4 (11)	34 (89)	0.727
High (> 12 yrs)	6 (7)	76 (93)	
Health Literacy			
Low	4 (31)	9 (69)	0.019*
Moderate	3 (5)	56 (95)	
High	2 (6)	29 (94)	
Computer Experience			
Low	6 (20)	24 (80)	0.037*
Average	2 (5)	42 (95)	
High	2 (4)	44 (96)	

(*) denotes statistically significance at p= <0.05

**Table 6:** Multivariable logistic regression model for tablet acceptance, help needed for questionnaire completion, and finding tablet questionnaires tiring

	Did not find electronic data capture acceptable OR (95% CI)	Needed help to complete questionnaires OR (95% CI)	Found electronic data capture tiring OR (95% CI)
Age	Reference: ≤ 70 years		
> 70 years	1.01 (0.96, 1.08)	1.01 (0.97, 1.06)	1.01 (0.96, 1.07)
Sex	Reference: Male		
Female	0.67 (0.13, 3.42)	1.37 (0.39, 4.77)	0.20 (0.10, 4.16)
Ethnicity	Reference: Caucasian		
Non-Caucasian	0.71 (0.14, 3.76)	2.84 (0.73, 11.10)	4.62 (0.79, 27.19)
Education	Reference: >12 years		
< 12 years	0.42 (0.07, 2.56)	1.03 (0.27, 3.95)	0.59 (0.10, 3.40)
Computer Experience	Reference: Average/above average		
Low	6.19 (1.06, 36.21)*	5.17 (1.34, 19.97)*	4.38 (0.78, 24.50)
Health Literacy		Reference: Moderate/high	
Low	0.53 (0.06, 4.59)	8.65 (1.77, 42.40)*	4.36 (0.78, 24.50)

Abbreviations: Odds Ratio (OR), Confidence Interval (CI)

Note: (*) denotes statistically significance at p= <0.05

## Discussion

Our main finding is that the tablet computer-based electronic data capture system was acceptable for the overwhelming majority of the enrolled patients with CKD. Although a majority of the study sample responded to the tablets positively, it appears that patients who are older (>70 years) with low health literacy and little previous experience with computers required more assistance while completing the questionnaires on the computer tablet.

Our results are qualitatively similar to the findings reported by Schick-Makaroff et al. [47, 48] whose study demonstrated that the electronic capture was overall positively received and viewed by patients. Similarly, studies from Harrington et al. [53] suggested that tablet computer platform is a feasible solution for monitoring and optimizing care of patients undergoing continuous ambulatory peritoneal dialysis [53]. In another study, tablet technology was also acceptable for nutrition monitoring, to support diet and fluid intake self-management, and medication inquiry in patients with CKD [54].

Although patients included in this study have CKD, similarly to the above studies, the severity of the condition and the different treatment modalities constitute an important difference. Patients on maintenance, in centre hemodialysis treatment are usually older, have more comorbidities and may be less educated than kidney transplant recipients or patients who are treated with home dialysis. Our patient population is also different from oncology patients, as we have older patient population groups and those with lower education. The chronicity if CKD/ESKD is also an important difference between many groups of oncology patients and our patient population. With psychosocial distress being a very frequent, currently largely neglected factor determining overall well-being of the patients, effective and reliable routine measurement of PROMs has the potential of detecting distress among patients with CKD and providing appropriate, tailored support when needed.

In our manuscript we provided data about the acceptability of tablet based collection of PROMs in in-centre dialysis patients and in kidney transplant patients. In addition to the papers of Schick-Makaroff, we used multiple questionnaire batteries in a heterogeneous patient population and found high acceptance and completion rates. At our institution the electronic data capture platform we used has already been linked to the electronic patient record system, providing opportunities for future clinical implementation. In our future work we will design specific implementation studies to establish the platform used in this pilot study for clinical use.

When considering electronic data capture to systematically assess patient reported outcome measures for either research or for clinical use it is important that there seems to be equivalency between completing questionnaires on touch-screen electronic capture platforms and paper-and-pencil administrations, at least for several of the instruments studies [48, 53, 54]^.^

The study is the first of its kind to investigate different patient populations with CKD, and also patients undergoing maintenance in center hemodialysis and kidney transplant recipients. In this pilot study we explored the use of multiple patient report instruments that assess various aspects of psychosocial characteristics of the patients. There was no meaningful difference in the acceptability of the various questionnaires in our study. Although the study was conducted in an academic teaching hospital, the patient population we encountered was diverse, both geographically and culturally.

An important first step in the routine incorporation of “ePROMs” in clinical practice is the establishment of the feasibility of tablet use among patients with CKD. Collection of ePROMs has the potential to increase physician patient communication [42, 44], integrate health-related quality of life (HRQOL) data into electronic patient records [41], screen for psychosocial distress and social difficulties [49]_,_ and promote the use of tailored educational materials specific to the needs of the patient at any given time [39, 42].

Several factors may have contributed to the high level of patient satisfaction. First of all, the research staff played an essential role in facilitating tablet use. Approximately one quarter of the participants reported none or very little computer experience, and required initial demonstration of the use of the tablet from the research staff. As tablet technologies become commonly used even by older people, we anticipate that patients will require less assistance given the greater familiarity with electronic devices. Second, in order to decrease respondent burden, the ePROM questionnaires were broken down into subsets with an average completion time of about 30 min. For many individuals with advanced medical illness, choosing select ePROM surveys tailored to the patient’s specific concerns could significantly reduce the response burden. Furthermore, utilizing electronic data capture platforms on handheld devices to assess PROMs will enable the use of computer adaptive testing algorithms, like the ones developed by patient-reported outcomes measurement information system (PROMIS), to increase measurement accuracy while decreasing patient response burden [55].

Our study has several limitations. This was a single centre study with convenience sampling and relatively few participants; this may limit generalizability of our results. Patients with language barrier, acute medical conditions or cognitive impairments were excluded from our study. Furthermore, participants with physical difficulties or visual impairments required assistance from the research staff in completing the questionnaires. With the diverse levels of disabilities, patients’ comfort towards technology will vary across different populations. If incorporated into routine clinical practice, appropriate support (from the clinical team or from trained volunteers) will be necessary to make this system feasible. In this pilot we did not use a control group to compare the acceptance of paper based versus electronic data collection. In fact, we wanted to assess if the electronic data capture platform is acceptable, feasible in the target patient population, in preparation for clinical implementation studies. Furthermore, we did not use qualitative methods to assess patient (or provider) attitudes and response to the tablet based electronic data capture system; we only wanted to focus on general acceptance of the system and difficulties with tablet use. We will include qualitative process evaluation methods in our future clinical implementation pilots of tablet based assessment of patient reported measures.

In addition, our sample size was small. Findings will need to be confirmed within a larger studies in the future. Finally, systematic assessment of patient PROMs in the clinical setting will necessitate an appropriate response system [49].

Results from this study suggested that the use of the tablet computer based electronic data capture system is acceptable for most patients and could be utilized to measure PROs among patients with CKD, both in hemodialysis and post-transplant clinics. Future studies will be needed to assess the larger scale, systematic use of such systems in routine clinical practice. In addition, individualized support and personal guidance should be emphasized among elderly patients and those with a visual or physical disability to assist with their functionality in using the tablet computer device. This practical and feasible approach could greatly enhance standard symptom monitoring and symptom management (including self-management) to improve quality of life and other clinical outcomes of patients as well as provide data for research and quality improvement to enhance care in any patient populations.

## Conclusion

Tablet-based electronic data capture system was an acceptable and feasible means of assessing PROMs among patients with chronic kidney disease. Patients who were elderly, or had little computer experience expressed lower acceptability for tablet-based technologies, and special consideration should be given in these populations.

## Additional file

Additional file 1: Patient response questionnaire (DOCX 18 kb)

## Abbreviations

CI: Confidence interval
CKD: Chronic kidney disease
CPC: Canadian problem checklist
DART: Distress assessment and response tool
DDKT: Deceased donor kidney transplantation
ECR: Experience in close relationship
ESAS: Edmonton symptom assessment system
GAD: Generalized anxiety disorder
GFR: Glomerular filtration rate
IIRS: Illness intrusiveness rating scale
KDQoL-SF: Kidney disease quality of life – short form
LDKT: Living donor kidney transplantation
MOS: Medical Outcomes Study
OR: Odds ratio
PHQ: Patient health questionnaire
PROM: Patient reported outcome measure
PROMIS: Patient-reported outcomes measurement information system
PRQ: Patient response questionnaire
RQ: Relationship questionnaire
SDI: Social difficulties inventory
SLS: Short literacy survey
TDMS: Transplant decision making survey
