# Supplementary material for: Exploring the use of tablet computer-based electronic data capture system to assess patient reported measures among patients with chronic kidney disease: a pilot study
Source: BMC Nephrol. 2017 Dec 6;18:356. doi: 10.1186/s12882-017-0771-7 (PMC5719517; doi:10.1186/s12882-017-0771-7)
Supplement: Additional file 1: — Patient response questionnaire (DOCX 18 kb) [file 12882_2017_771_MOESM1_ESM.docx]

# Patient Response Questionnaire

1. Did you find the task of completing the questionnaires ON THE TABLET COMPUTER ACCEPTABLE?

- - Yes
  - No

2. Did you need someone’s help to complete the questionnaire?

- - Not at all
  - Very little
  - Somewhat
  - A great extent
  - Someone completed it for me

1. Did you find the questions acceptable to you?
   - Yes
   - No

4. Did you find the task of completing the questionnaires ON THE TABLET COMPUTER too difficult or tiring?

- - Yes
  - No

5. How much computer experience do you have?

- - None
  - A Little
  - Average
  - Somewhat above average
  - A Lot
  - An exceptional amount

6. How comfortable are you in using computer technology generally?

- - Very uncomfortable
  - uncomfortable
  - fair
  - good
  - very comfortable
  - excellent
